# A pyroptosis-related signature in colorectal cancer: exploring its prognostic value and immunological characteristics

**DOI:** 10.7717/peerj.16631

**Published:** 2023-12-19

**Authors:** Peicheng Jiang, Jin Fan, Shenglin Huang, Luying Liu, Minghua Bai, Quanquan Sun, Jinwen Shen, Na Zhang, Dong Liu, Ning Zhou, Yanru Feng, Jin Jiang, Ji Zhu

**Affiliations:** 1 Department of Radiation Oncology, Zhejiang Cancer Hospital, Hangzhou, China; 2 Hangzhou Institute of Medicine (HIM), Chinese Academy of Sciences, Hangzhou, China; 3 Department of Radiation Oncology, Fudan University Shanghai Cancer Center, Shanghai, China; 4 Fudan University Shanghai Cancer Center and Institutes of Biomedical Sciences Fudan University, Shanghai, China; 5 Department of Radiation Oncology, The First Hospital of Jiaxing Affiliated to Jiaxing University, Jiaxing, China; 6 Jiaxing Key Laboratory of Radiation Oncology, 2019 Jiaxing Key Discipline of Medicine, Jiaxing, China; 7 Zhejiang Key Laboratory of Radiation Oncology, Hangzhou, China

**Keywords:** Pyroptosis, Colorectal cancer, Prognosis, Immunological characteristics, personalized treatment

## Abstract

**Background:**

The heterogeneity of colorectal cancer (CRC) is the main cause of the disparity of drug sensitivity and the variability of prognosis. Pyroptosis is closely associated with the development and prognosis of various tumors, including CRC. Dividing CRC into distinct subgroups based on pyroptosis is a worthwhile topic for improving the precision treatment and prognosis prediction of CRC.

**Methods:**

We classified patients into two clusters using the consensus clustering based on the pyroptosis-related genes (PRGs). Next, the prognostic signature was developed with LASSO regression analysis using the screened genes from differentially expressed genes (DEGs) by univariate and multivariate Cox analyses. According to the pyroptosis-related score (PR score) calculated with the signature, patients belonged to two groups with distinct prognosis. Moreover, we assessed the immune profile to explore the relationship between the signature and immunological characteristics. Two single cell sequencing databases were adopted for further exploration of tumor immune microenvironment (TME). In addition, we applied our own cohort and Drugbank to explore the correlation of the signature and clinical therapies. We also studied the expression of key genes by immunohistochemistry.

**Results:**

The signature performed well in predicting the prognosis of CRC as the high area under curve (AUC) value demonstrated. Patients with a higher PR score had poorer prognosis and higher expression of immune checkpoints but more abundant infiltration of immune cells. Combining with the indicator of therapeutic analysis, they might benefit more from immune checkpoint blockade (ICB) and neo-adjuvant chemoradiotherapy (nCRT).

**Conclusion:**

In conclusion, our study is based on genomics and transcriptomics to investigate the role of PRGs in CRC. We have established a prognostic signature and integrated single-cell data to study the relationship between the signature with the TME in CRC. Its clinical application in reliable prediction of prognosis and personalized treatment was validated by public and own sequencing cohort. It provided a new insight for the personalized treatment of CRC.

## Introduction

Colorectal cancer (CRC) is a type of cancer that is a significant cause of morbidity and mortality. According to the American Society of Clinical Oncology, the global morbidity of CRC is rising in people under 65 years of age. Although its overall morbidity has declined due to widespread screening, it is estimated that the deaths caused by CRC will be the third most common in 2021 (Siegel et al., 2021). Despite the improvements in the treatment, the 5-year survival rate for metastatic CRC is approximately 14% (Smith et al., 2019).

Pyroptosis, distinct from other programmed cell death, involves the activation of gasdermin (GSDM) family proteins as the effector molecules. Activated GSDMs translocate to the cell membrane, leading to the membrane pores. The resulting osmotic disparities between the intracellular and extracellular environments cause continuous cellular swelling, culminating in cell rupture and death, accompanied by the release of intracellular contents and initiation of inflammatory responses (Galluzzi et al., 2018). In the classical pyroptosis pathway, inflammasomes recruit and activate Caspase-1, which cleaves the N-terminal of GSDMD, enabling its binding to the membrane and facilitating pore formation. Caspase-1 also cleaves pro-inflammatory cytokines IL-1β and IL-18, promoting their maturation and release (Yu et al., 2021). As researchers’ last interest in pyroptosis, several non-classical pyroptotic pathways have been discovered, such as those dependent on caspase-4, caspase-5/11 (Shi et al., 2014; Ding & Shao, 2017; Yi, 2017), and the induction pathway mediated by Granzyme A, a protease secreted by cytotoxic lymphocytes that cleaves GSDMB (Zhou et al., 2020). Recent studies have also revealed the association between the tumor immune microenvironment (TME) and neoadjuvant chemoradiotherapy (Chatila et al., 2022; Wen et al., 2022).

The intricate association between tumorigenesis and chronic inflammation has been well-established (Chang & Yang, 2016). The induction of pyroptosis contributes to heightened inflammatory infiltration, creating a microenvironment conducive to tumor initiation and metastasis (Balkwill & Mantovani, 2001). In recent years, pyroptosis has garnered extensive attention in cancer research (Wu et al., 2021; Fang et al., 2020). The elevated expression of members of the GSDM family in tumor tissues has been found to correlate with aggressive tumor behavior and unfavorable prognosis in various malignancies (Sun et al., 2022; Gao et al., 2018; He et al., 2021). However, it is noteworthy that pyroptosisis is a dual-edged sword, as it also exhibits anticancer effect (Chen et al., 2015). It also has a profound influence on immune cell infiltration within the tumor microenvironment (TME), and identifying the role of pyroptosis in tumor immunity has been fruitful. A study revealed that GSDM-mediated pyroptosis could enhance the antitumor immunity through a cytotoxic lymphocyte-killing mechanism (Zhou et al., 2020). A small proportion of cells undergoing pyroptosis can efficiently regulate the tumor immune microenvironment and facilitate an antitumor immune response (Wang et al., 2020; Wang et al., 2017). GSDME-mediated pyroptosis triggers the release of HMGB1, which in turn activates the ERK1/2 pathway, effectively promoting the polarization of M2 macrophages of CRC (Tan et al., 2020; Mu et al., 2018). Notably, the synergistic administration of BRAF inhibitors and MEK inhibitors facilitates the recruitment of dendritic cells and activated T cells, accomplished through the heightened cleavage of GSDME and subsequent release of HMGB1 (Erkes et al., 2020). GSDME-mediated pyroptosis leads to an augmented infiltration of natural killer (NK) cells and CD8+ T lymphocytes and induces phagocytosis by tumor-associated macrophages, further contributing to the anti-tumor response (Chao et al., 2008). Recent studies have also revealed the association between the tumor immune microenvironment (TME) and neoadjuvant chemoradiotherapy (nCRT) (Chatila et al., 2022; Wen et al., 2022). However, there is a lack of exploration regarding the relationship between pyroptosis and nCRT.The exploration of pyroptosis in CRC has provided initial insights, yet the underlying mechanisms remain elusive, with a dearth of comprehensive profiling. The expression of GSDMD, a marker of pyroptosis, is significantly downregulated in human CRC tissues, and its expression negatively correlates with the prognosis of CRC (Wu et al., 2021; Fang et al., 2020). The expression of NALP1, a pyroptosis inducer, is lower in tumor tissues than in paratumoral tissues (peritumoral and adjacent healthy tissues) and is linked to tumor metastasis and survival of CRC. DAC (5-aza-2-deoxycytidine) hinders the growth of CRC and increases the lifespan by restoring NALP1 levels (Chen et al., 2015). While Li et al. (2022) constructed a pyroptosis-related prognostic model with bioinformatics analysis, the relatively low AUC value of the model indicated that its predictive performance was unsatisfactory. The pyroptosis-related robust signature we developed in this study provides a novel perspective on the identification the prognosis of CRC. The signature facilitates to assess the feasibility of immunotherapy and the sensitivity of chemotherapy in personalized CRC management.

## Materials and Methods

**Figure 1:**
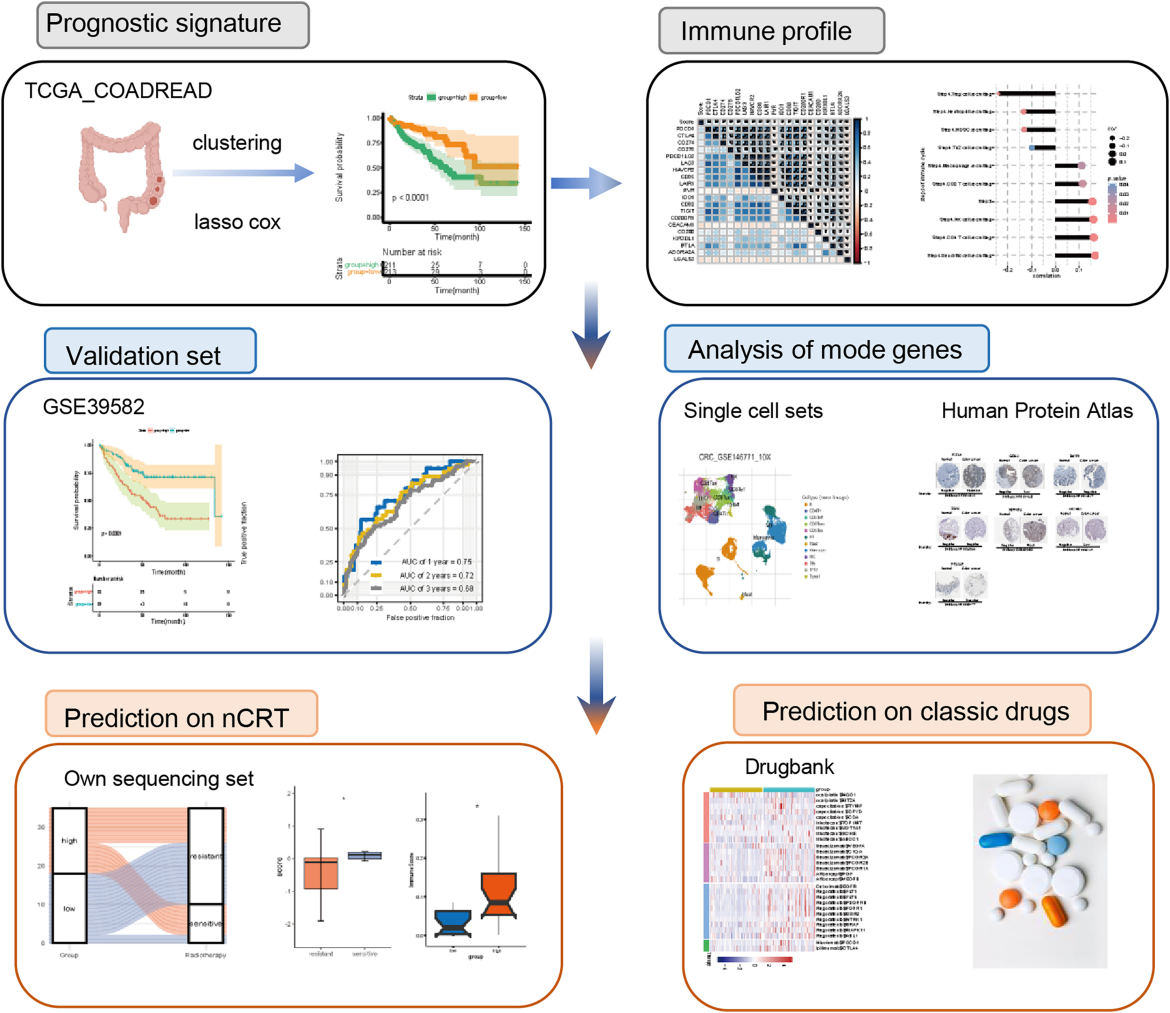
Flowchart of the study. nCRT, neo-adjuvant chemoradiotherapy

The flowchart was of this study was presented in Fig. 1.

### Dataset collection and pre-processing

RNA sequencing (RNA-seq) data (count values) and simple nucleotide variation in patients with CRC and the relevant clinical characteristics were obtained from TCGA dataset (https://portal.gdc.cancer.gov). The transcriptomic data of healthy colon tissues were retrieved from the GTEx database (http://xena.ucsc.edu/). To enable comparison with the GTEx dataset, we normalized the count values from both datasets using log2 (X + 1) transformation. For the accuracy of the functional analysis, we transformed the count value to tpm (transcripts per million) in the subsequent analyses. We excluded the cases lacking the survival information and cases with vague survival information. The somatic mutation data of patients with CRC was used to analyze the mutation profiles using the “maftools” package. MSI data were acquired from the cBioPortal website (https://www.cbioportal.org/).

The GSE39582 dataset from GEO (http://www.ncbi.nlm.nih.gov/geo) was chose as the external validation cohort. The samples in the validation set have survival information and their tumor stages are similar to those in the training set (Table S1). The single‑cell transcriptomic datasets GSE14677, GSE13639 were acquired from the Tumor Immune Single-cell Hub (TISCH).

The Institutional Review Boards of Zhejiang Cancer Hospital granted approval (No. IRB-2021-291) for this study, which was conducted in accordance with the ethical guidelines outlined in the Declaration of Helsinki. A total of 35 patients’ tissues of rectal cancer and their clinical infromation were collected from Zhejiang Cancer Hospital. All participants signed informed consent according to the Institutional Review Boards of Zhejiang Cancer Hospital. All tissue were stored at −80 °C once dissected from patients until sequencing. Total RNA was extracted from the CRC tissues. After assessing the quality and integrity of the RN, the enriched mRNA was fragmented into small pieces and used as a template for cDNA synthesis with random primers. The second-strand cDNA was synthesized, followed by end repair, A-tailing, and adapter ligation. The ligated products were amplified by PCR to construct the cDNA libraries. The libraries were quantified using qPCR and sequenced on an Illumina HiSeq platform. The read counts for each gene were obtained using HTSeq (Illumina Corporation, Foster City, CA, USA).

### Classification of patients based on pyroptosis patterns

Thirty-three PRGs were extracted from prior studies (Duan et al., 2016) are shown in Table S1. On the basis of PRGs, we implemented consensus clustering to identify distinct PR patterns using the k-means method. We performed it with the “ConsensuClusterPlus” package and set the parameter “repetition” as 1,000 for stability (Wilkerson & Hayes, 2010).

### Functional enrichment analysis of the DEGs

We adopted the “DESeq” package to identify DEGs between different PR patterns, setting the significance criteria for differential expression at *P* < 0.05 and an absolute Log2 fold change (FC) >1. We applied the “clusterProfiler” package (Yu et al., 2012) for functional enrichment analysis of DEGs. We retrieved immune-related genes from the Gene Ontology database (http://geneontology.org/) and performed ssGSEA to assess differences between clusters in immune-related pathways. The hallmarker genesets were obtained from the Molecular Signatures Database (MSigDB) (https://www.gsea-msigdb.org/gsea/msigdb/index.jsp) for Gene Set Variation Analysis (GSVA).

### Establishment of the pyroptosis-related prognostic model

To evaluate the survival significance of the DEGs, we utilized univariate and multivariate Cox regression analyses. Thirty-four genes (*P* < 0.05) were selected for the next analysis. To screen optimal genes, the ‘glmnet’ package was applied to carry out the LASSO Cox regression analysis (Duan et al., 2016). Then, we used the ten selected genes and their corresponding coefficients to construct a prognostic signature. The score based on the pyroptosis signature was calculated using the subsequent equation: PR score = 0.112 ×Expr_*FCRL1*_ + 0.252×Expr_*WNT16*_ + 0.130×Expr_*GRIK2*_ + 0.070×Expr _*ZMAT1*_ – 0.005×Expr_*ZG16*_ + 0.103×Expr_*DRD4*_ + 0.044×Expr_*MAPK12*_ + 0.220×Expr_*OR51B5*_ + 0.004×Expr_*PRSS21*_ + 0.004×Expr_*MAGEA3*_, where Expr_i_ is the gene expression level. Utilizing the median risk score as a threshold, we categorized patients into the high-score group and low-score group. The interaction network was mapped using Cytoscape (version 3.8.0) (Shannon et al., 2003).

### Assessment of the immunological characteristics

We evaluated the immunological profile of CRC patients, including inhibitory immune checkpoints, cancer immunity cycle activity, and immune cells infiltration. The genes regulating the clinical response to ICBs are characterized from 20 solid cancers and established The Cancer Immunome Atlas (TCIA) (Auslander et al., 2018).

Comprising seven steps, the cancer immunity cycle reflects the intricate interplay between the processing of neoantigens and the prevention of autoimmunity (Chen & Mellman, 2013). To present TIICs precisely, we assessed the infiltration status by applying several independent algorithms comprehensively, including CIBERSORT, TIP, and xCell (Chen & Mellman, 2013; Aran, Hu & Butte, 2017). In addition, we performed single-cell analysis to explore the correlation of key genes and TME with TISCH (Sun et al., 2021).

### Analysis of chemotherapy drugs and nomogram construction

DrugBank is a publicly available database that integrates bioinformatics and medicinal chemistry to provide information on drugs and their targets (Uhlén et al., 2015).

For applying the signature in clinical settings, we used the key genes of the signature to construct the nomogram using ‘rms’, ‘nomogramEx’, and ‘regplot’ packages.

### The levels of key proteins in clinical specimens

The Human Protein Atlas (HPA, version: 18.1) (https://www.proteinatlas.org/) aims to provide a public platform for researchers to explore the gene expression landscape of 24,000 human proteins (Uhlen et al., 2017). We explored the the protein expression of key genes applying the HPA database.

### Statistical analysis

Correlation coefficients were calculated using Pearson chi-square test.

We used the Kaplan-Meier method from the “survival” package to generate survival curves of which statistical significance was estimated with log-rank tests. The time-dependent ROC curves with AUC values were created using “pROC” and the “time ROTC” packages. The Wilcoxon test and Kruskal-Wallis test were utilized to examine the differences between two groups and multiple groups, respectively. All statistical analyzes were performed with R (R Core Team, 2023). The statistical threshold for significance was *P* < 0.05.

## Results

### Expression of PRGs in CRC

**Figure 2:**
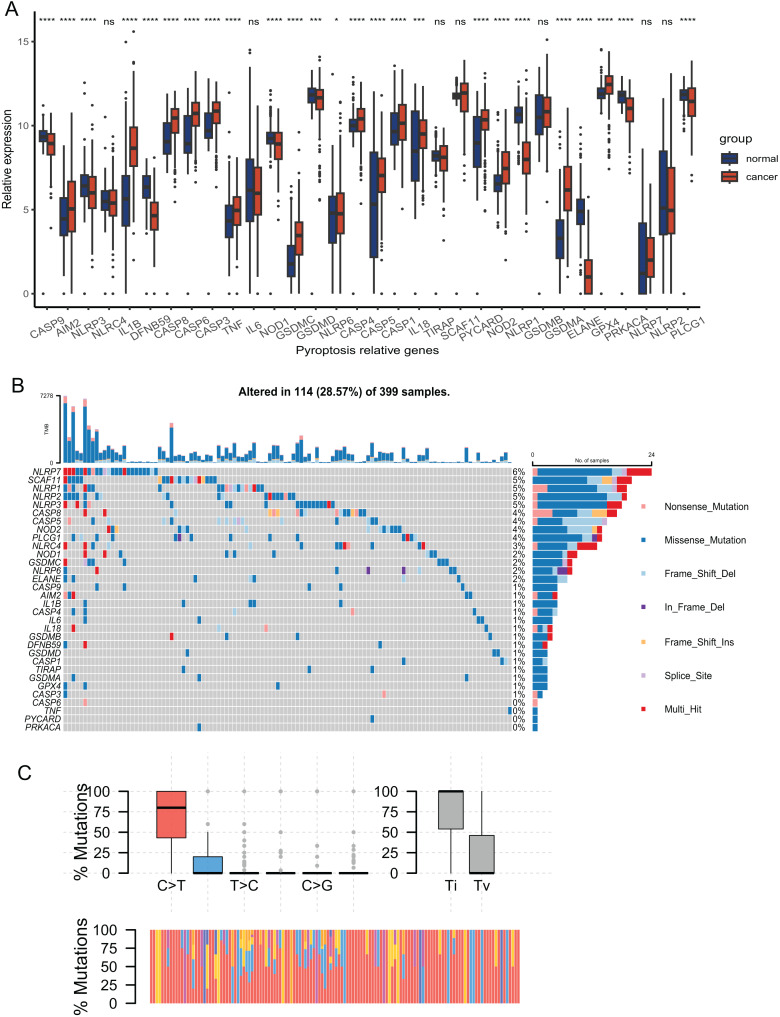
Expression and mutation profiles s of the 33 pyroptosis-related genes. (A) Expression of the pyroptosis-related genes between the normal and the tumour tissues. *P* values were showed as: *P < 0.05; ***P < 0.001; ****P < 0.0001. (B) Mutation information of each PRGs in the waterfall plot. Colors with annotations at the bottom mean different mutation types. The barplot showed mutation burden. (C) Types of SNP. C > T was the most common of single-nucleotide variant and Ti showed higher proportion than Tv. Ti, transition; Tv, transversion.

The expression of 33 PRGs were compared between the tumor and normal tissues after pre-processing the data from TCGA and GTEx. The results demonstrated that the disparities in the expression of 27 PRGs were significant (Fig. 2A). To explore the mutation profiles of PRGs in CRC, we analyzed somatic mutation data using the VarScan2 Annotation. The mutation information of the PRGs showed that 114 patients had pyroptosis-related regulator mutations (Fig. 2B). The gene with the highest frequency of mutations was NLRP7, and the mutation rate of the five genes was up to 5%. C-T was the predominant single-nucleotide variation in CRC (Fig. 2C). The high proportion of differentially expressed PRGs and the mutation profile implied that pyroptosis played a vital role in CRC.

### Identification of a classification pattern mediated by PRGs in CRC

**Figure 3:**
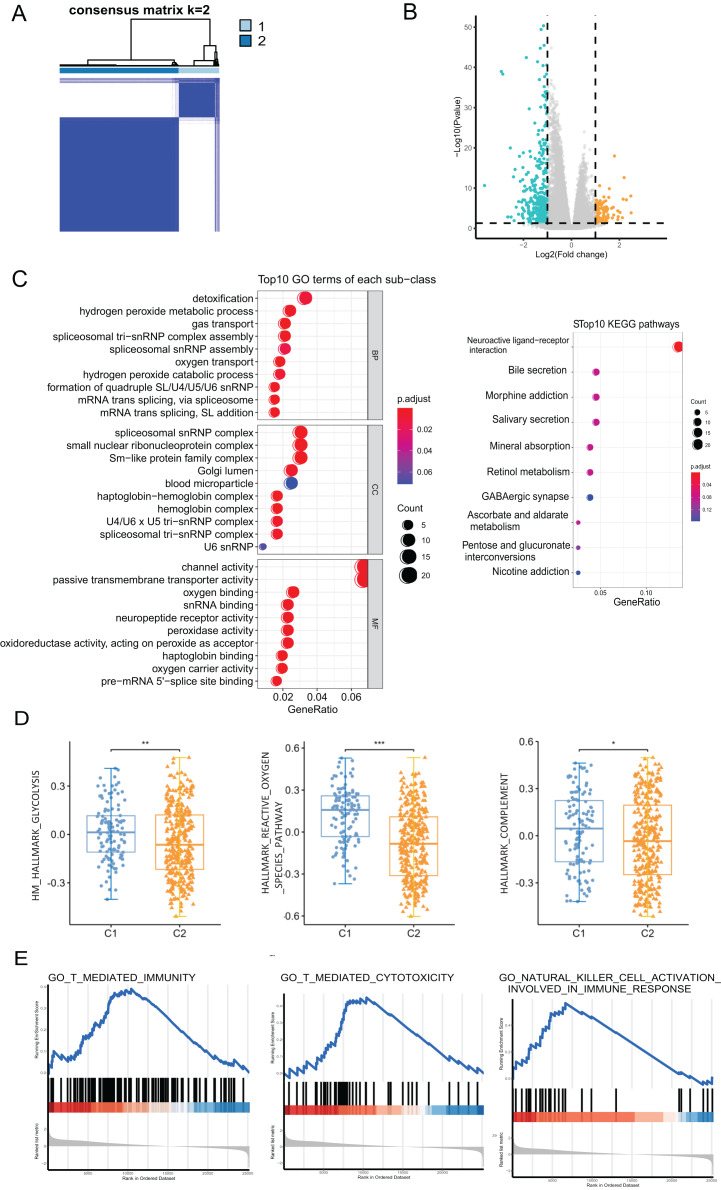
The analyses to DEGs of two pyroptosis-related clusters. (A) A total of 424 patients were grouped into two clusters according to the consensus clustering matrix (k = 2). (B) The expression of DEGs between two clusters. (C) GO enrichment and KEGG pathways analyses for PRGs. (D) The profile of classic characteristics of the two clusters based on hallmarker genesets. (E) Gene enrichment analysis for PRGs based on immune geneset from the GO database. *P < 0.05; **P < 0.01; ***P < 0.001.

We performed a consensus clustering analysis of CRC patients based on PRGs in TCGA cohort. The results showed that two different regulation patterns were identified, including 106 cases in cluster 1 (C1) and 318 cases in cluster 2 (C2) (Fig. 3A). To explore the biological differences between the two pyroptosis-related clusters, we performed a series of analyzes. First, the differential analysis was performed to identify 489 DEGs. The differential expression of DEGs was visualized using a volcano plot (Fig. 3B).

Enrichment analyses for GO and KEGG demonstrated that the DEGs were enriched in hydrogen peroxide metabolism, spliceosome snRNP complex, and channel activity (Fig. 3C). To profile the characteristics of the two clusters, we performed GSVA analysis using the hallmark genesets. The analysis revealed significant differences in various aspects, including metabolism, stress response, and immune between the two clusters. For instance, disparities were observed in pathways such as glucose metabolism, reactive oxygen species pathway, and the complement system (Fig. 3D and Fig. S1A). Considering the association of pyroptosis and immunity, we used GSEA with the annotation of the immune system (GO:0002376) to investigate the differences in the immunity of the two pyroptosis-related clusters. The enrichment of various pathways presented significant differences, including natural killer cell activation, T cell-mediated immunity, antigen processing and presentation, *etc*., (Fig. 3E and Fig. S1B).

### Construction and verification of the pyroptosis-related prognostic signature

**Figure 4:**
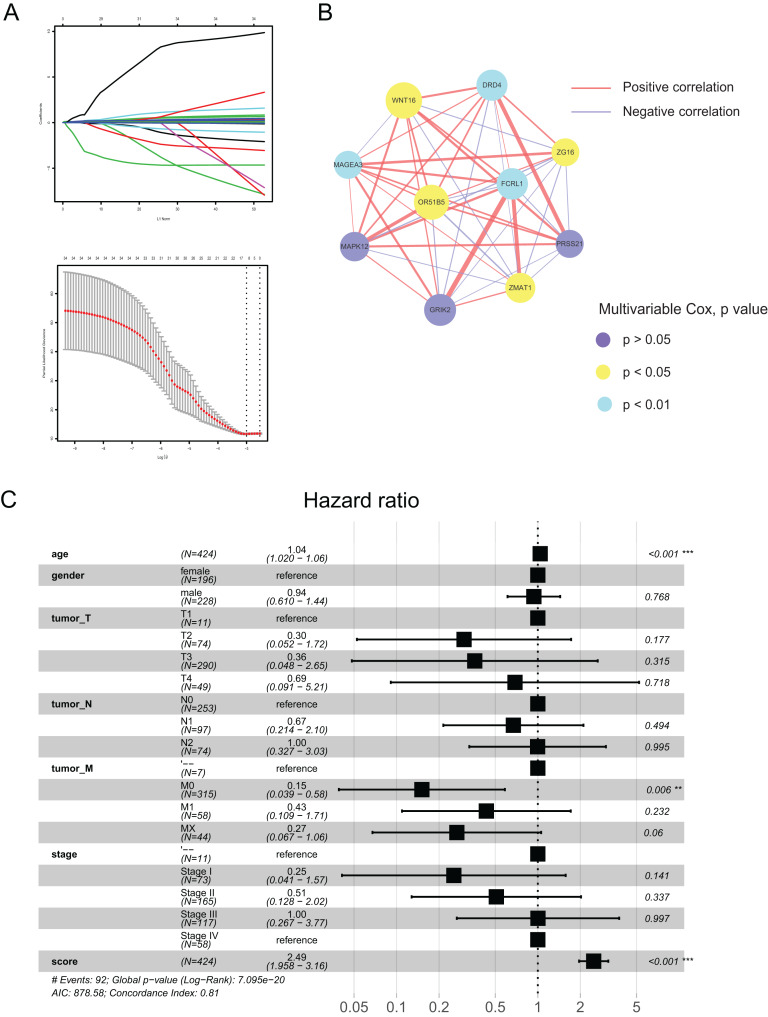
Construction of a prognostic signature based on pyroptosis-related clusters. (A) In the LASSO-Cox model. Adopting the minimum standard to acquire the value of the super parameter l by 10-fold cross-validation. (B) The interaction network of 10 key genes. The line size and the dot size are positively with correlation and hazard ration, respectively. (C) Forest plot of multivariate Cox regression analysis between PR score and clinical characteristics. **P < 0.01; ***P < 0.001.

Using the differences in the two pyroptosis-related patterns, we built a prognostic model and validated its stability and veracity as follows. First, DEGs were screened using the univariate and multivariate Cox regression analyses for prognostic prediction. Next, we narrowed down the candidate genes by applying the LASSO Cox regression model with a minimum penalty parameter (λ) (Fig. 4A). Ultimately, we obtained 10 key genes with which we developed a pyroptosis-related prognostic model. With this signature, each patient could obtain a corresponding score named the pyroptosis-related (PR) score. We then performed multivariate Cox analysis on key genes to reveal their impact on prognosis. (Fig. S1). In Fig. 4B, we presented the interaction network between key genes,which was constructed based on gene co-expression patterns.

To assess the prognostic value of PR score, univariate and multivariate Cox regression analyses were performed between it and clinical characteristics. The hazard ratio (HR) of PR score was 2.49 (95% CI [1.958–3.16]; *P* < 0.001), indicating that PR score could serve as an independent prognostic factor in CRC (Fig. 4C).

**Figure 5:**
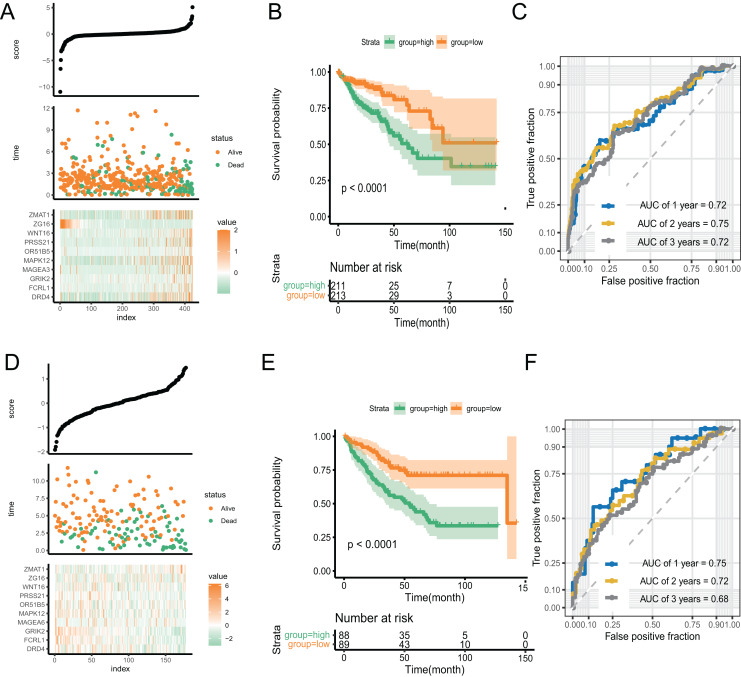
The discriminatory ability estimation of the signature in training and validation cohort, respectively. (A and D) Distribution of patients and key genes based on the risk score. (B and E) Kaplan–Meier curves of patients in the high- and low-score groups. (C and F) The time-dependent ROC analysis of the PR score. Abbreviations: AUC, area under the curve; ROC: receiver operating characteristic.

With an increase in the PR score, the survival status of patients and expression of key genes exhibited significant differences. (Fig. 5A). We performed Kaplan–Meier and time-receiver operating characteristic curves (ROC) analyses to assess the discriminatory performance of the prognostic signature. The analysis suggested that patients in the high-score group had worse prognosis (*P* < 0.001) (Fig. 5B). The AUC for 1-, 2-, and 3-year in the ROC curve analysis was 0.72, 0.75, and 0.72, respectively, indicating excellent discriminatory ability of the prognostic signature (Fig. 5C). We validated the signature by applying it to GSE39582 dataset and performing Kaplan-Meier and time-ROC analyses. The results verified the robustness of the signature (Figs. 5D–5F).

### Tumor immunity-associated characteristics

GSEA of the DEGs revealed the differences in the enrichment of multiple immune-related pathways. To explore the utility of the signature in the tumor immune microenvironment, we performed several analyses to evaluate the relationship between the PR score and the characteristics of tumor immunity.

**Figure 6:**
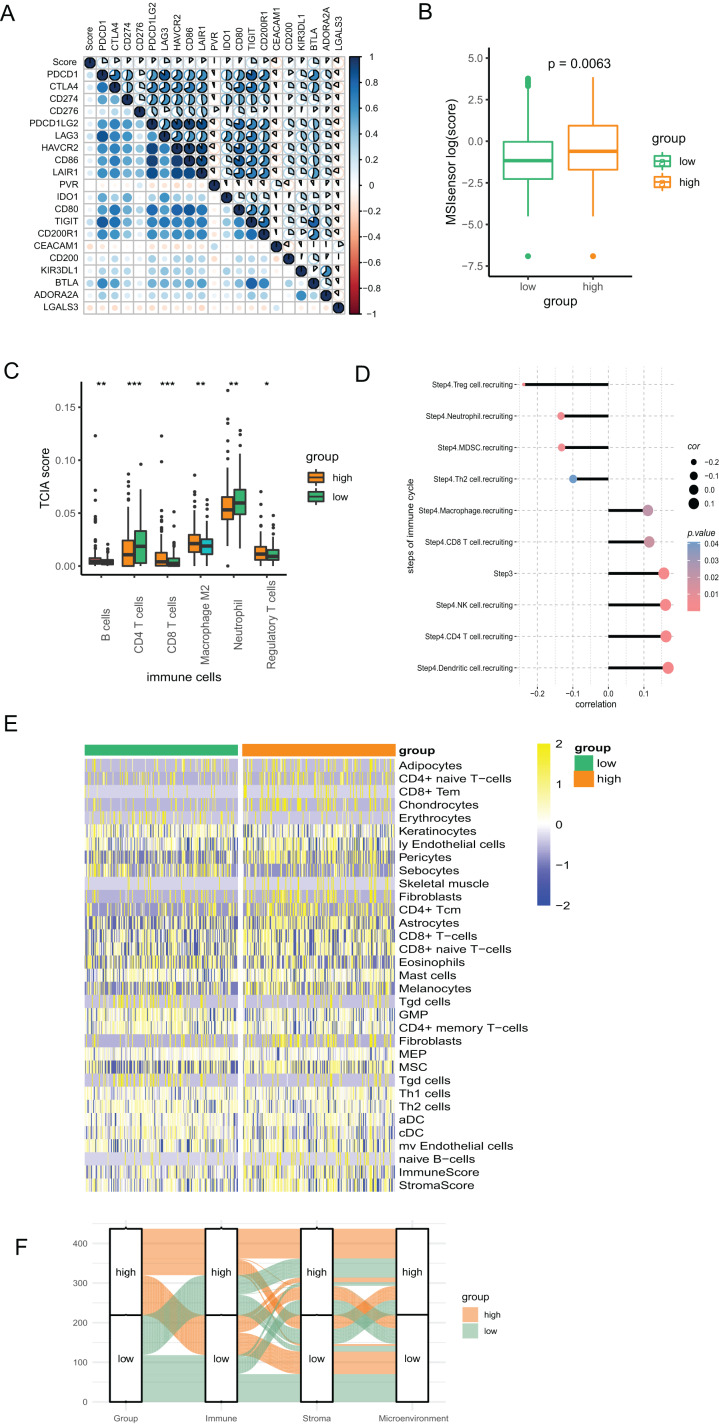
The association between the signature and tumor immunity associated characteristics. (A) The correlation of immune checkpoint genes and PR score. (B) The MSI score in two groups. (C) The differences of immune cells in two groups based on TCIA. (D) The correlation of steps in immune cycle and PR score (E) The infiltration of immune cells in two groups based on xCell. (F) Sankey plot displaying the coherence between PR score and immune score, matrix score, microenvironment score. *P < 0.05; **P < 0.01; ***P < 0.001.

We conducted Pearson correlation analysis to investigate the correlation of the PR score and the immune checkpoints. As presented in Fig. 6A, PR score was closely associated with various immune checkpoints, including PD1, PD-L1, and CTLA4, which is valuable in immunotherapy. Additionally, our study suggested that PR score may serve as an indicator of cancer immunogenicity in CRC, as demonstrated by the higher microsatellite instability (MSI) scores in the high-score group based on the results of Wilcoxon analysis (Fig. 6B). The above results indicated the role of PR score in immunotherapy. We combined the data of TCIA to analyze the correlation between PR scores and immune cells. The infiltration of multiple immune cells, including B cells, several subtypes of T cells, macrophage M2, neutrophils, and regulatory T cells (Treg) cells, was found to be significantly different between the two groups (Fig. 6C). Moreover, the abundance of B cells, CD8 T cells, dendritic cells, M2 macrophages, and neutrophils were strongly related to the PR score (Fig. S2). The immunity cycle sorts the process of antitumor immunity into seven steps, composed of 23 processes. Activities of multiple processes in the cycle were changed, including the priming and activation (Step 3) and infiltration of immune cells to tumors (Step 4) (CD8 T, CD4 T, macrophage, Th2, NK, and Treg cell recruitment) (Fig. S3). By further analyzing the correlation, we found that the PR score was related to the recruitment of various immune cells (Fig. 6D).

To assess the infiltration of TIICs with different algorithms for verification, we downloaded the data of immune cells from xCell based on the ssGSEA algorithm to investigate the variation as the PR score changes. The heatmap showed that the infiltration of multiple above-mentioned immune cells was associated with the PR score, which is consistent with the findings from TCIA. (Fig. 6E). The Sankey diagram suggested good coherence between the PR score and immune score, matrix score, and microenvironment score (Fig. 6F).

### Single-cell analysis for the key genes of the signature

**Figure 7:**
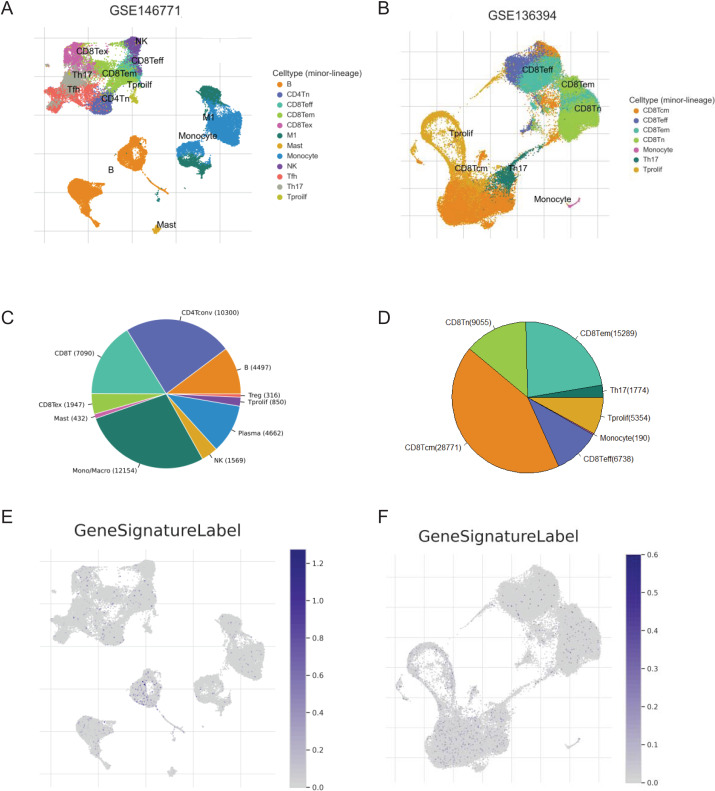
The correlation of key genes and tumor immune cells. (A and B) Cell types in GSE124765 and GSE139555. (C and D) The proportion of immune cells. (E and F) The expression of key genes in GSE124765 and GSE139555. CD4Tconv, conventional CD4 T cells; CD8Tn, naive CD8 T cells; CD8Tex, exhausted CD8 T Cells; CD8 Tem, effector memory T cells CD8 Tcm, central memory T cells; Tprolif, proliferating T cells.

We screened two scRNA-seq atlases of CRC from TISCH database to explore the immunological characteristics of the signature. The 10 samples of GSE14677 are tumor tissues from patients without therapy. And the five samples of GSE13639 are tumor tissues from patients who accepted adoptive cell therapy. The percentage of CD4Tconv cells was the biggest in untreated patients and the number of CD8Tcm cells was predominated in patients after immunotherapy (Figs. 7A–7D). To analysis the association of the signature and immune cells, we analysed the expression of genes with positive coefficient in cell clusters. The intense expression of these genes in B cells indicated positive relevance between PR score and the infiltration of B cell (Fig. 7E). That was consistent with the results of TCIA and xCell (Figs. 6C and 6E). By contrast, the expression of these genes presented different distribution features in GSE13639. The level of these genes was highest in CD8Tcm cells (Fig. 7F). The difference should be caused by adoptive cell therapy and it indicated the interaction between immunotherapy and PR score.

### Potential clinical application of the signature

**Figure 8:**
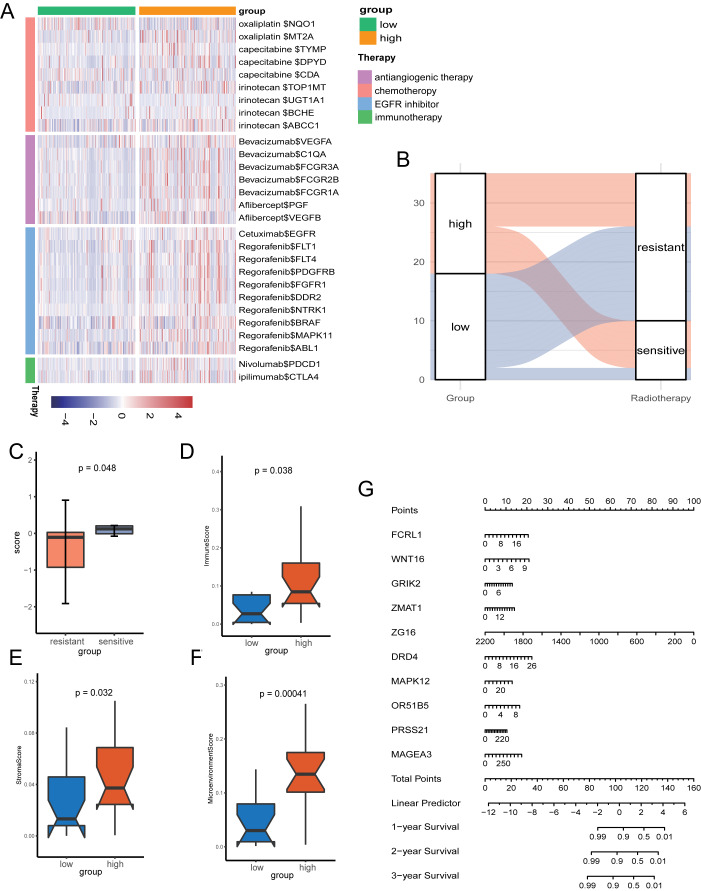
The potential clinical application of the signature. (A) Correlation between PR score and the colon cancer-related drug-target genes screened from the Drugbank database. (B) Sankey plot displaying the coherence between the signature and the sensitivity of nCRT. (C) The differences of PR score in two groups. (D–F) The TME of two groups in nCRT cohort. (G) Nomogram for predicting 1, 3, and 5 years overall survival for colon cancer patients.

Given the role of pyroptosis in oncotherapy, we extracted the target genes in CRC from the Drugbank database and studied the association between the PR score and the target genes. Figure 8A showed the differences in the expression of many genes targeted by drugs, such as oxaliplatin, irinotecan, bevacizumab, nivolumab, and ipilimumab, suggesting different responses for two groups to chemotherapy, molecular targeted therapy, and immunotherapy.

Neo-adjuvant therapy (nCRT) palyed an irreplaceable role for CRC patients, particularly rectal cancer. For the exploration of nCRT with the signature, we analysis the the response of nCRT and the PR score. The result indicated that patients with higher PR scores were more likely to be sensitive to nCRT, while those with low scores were more likely to be resistant (Fig. 8B). The further analysis of score and the response of nCRT revealed that score of patients resistant to nCRT was significantly lower than the counterparts (Fig. 8C, *P* < 0.05). The evaluation of immune cells with xCell was consistant with the training set, namely, patients belonged to the high-score group had better infiltration of immune cells (Figs. 8D–8F).

Because of the value of the PR score in predicting the prognosis, we generated a nomograph featuring 10 key genes for the clinical management of patients with CRC (Fig. 8G). It would help clinical doctors in making personalized treatment efficient.

### The protein expression of key genes in human tissue

**Figure 9:**
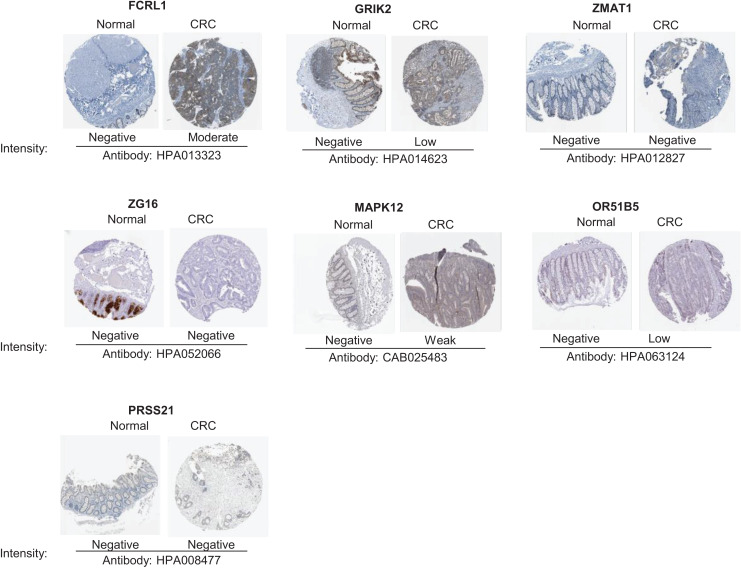
Verification of key genes expression in normal colon tissues and tumor tissues. CRC, colorectal cancer.

To validate the reliability of the prognostic signature at protein level, we investigated the expression of most key genes in human tissue samples with HPA database except for WNT16, DRD4 and MAGEA3, which were unavailable. As shown in Fig. 9, the protein expression of FCRL1, GRIK2, MAPK12, and OR51B5 showed differential level between normal colon tissue and tumor tissue (Fig. 9).

## Discussion

As a new type of non-apoptotic programmed cell death, pyroptosis has been extensively studied in recent years, revealing its potential as an anti-tumor mechanism in various cancers (Johnson et al., 2018; Williams et al., 2015). Although there have been several studies that have explored the relationship between pyroptosis and CRC (Ning et al., 2023; Hu et al., 2022; Li et al., 2022; Chen et al., 2022; Wei et al., 2021; Zhuang et al., 2021; Tan et al., 2020; Derangère et al., 2014). Our research employs a comprehensive approach. We have utilized genomics, transcriptomics, single-cell data, and our own sequencing data of nCRT. Furthermore, we have validated our findings using external datasets. This multifaceted approach allowed us not only to construct a robust prognostic model but also to delve into relevant biological features. However, its influence on the prognosis of CRC needs more exploration to elucidate. In this article, we studied the prognostic value of pyroptosis in CRC and constructed a well-performed and robust prognostic signature. Further exploration demonstrated its correlation with immunological characteristics. Additionally, leveraging our sequencing dataset, we conducted an in-depth analysis that uncovered the predictive value of pyroptosis in the context of nCRT sensitivity.

Cancer immunotherapy, focusing on the potent cytotoxicity of immune cells, has emerged as a compelling frontier in the field of oncology, owing to its distinctive mechanism and commendable clinical efficacy (Cable et al., 2021). ICBs were approved by the FDA in 2017 for CRC patients with mismatch-repair-deficient and high microsatellite instability score (dMMR–MSI-H) (Ganesh et al., 2019). However, a substantial challenge persists as patients with proficient mismatch repair, microsatellite stability, or low levels of microsatellite instability (pMMR-MSI-L) derive minimal therapeutic benefit from immunotherapy interventions (Overman et al., 2017). Therefore, there exists an urgent need to enhance the precision of prognostic assessment and expand the eligible subpopulation that can obtain the rewards of ICBs. Our analyses demonstrated the positive correlation between PR score and MSI score, which indicated the value of PR score on improving the accuracy of immunotherapy.

Given the intrinsic resistance of tumor cells to apoptosis, pyroptosis emerges as an alternative mechanism for anti-cancer therapy (Wang, Liu & Zhao, 2019; Huang et al., 2018). There is an investigation recently demonstrated that pyroptosis enhances the antitumor activity of immune checkpoint inhibitors (ICIs), even in ICB-resistant tumors (Tang et al., 2020). The analyses we conducted to explore the immunity-associated characteristics showed that the PR score is correlated with TIICs and immune checkpoints. The correlation between PR score and the expression of PD1 and CTLA4, the most known inhibitory immune checkpoints, were statistically significance. It reminded us of the potential of PR score to be predictor of ICBs. Wang et al. (2020) recently found that only 15% of tumor cells undergoing pyroptosis could lead to the death of the tumor graft. Another study found that pyroptosis facilitated the infiltration of CD8^+^ T cells and NK cells, synergizing the efficiency of ICBs. Consistent with this, our signature showed that the PR score was positively related to the infiltration of CD8^+^ T cells and NK cells and the expression of immune checkpoints, revealing that patients with higher scores may be more responsive to ICBs. This may be due to the higher abundance of immune cells in patients with high PR scores. However, the simultaneous higher expression of immune checkpoint inhibitors (ICIs) in these patients significantly suppresses the immune surveillance and cytotoxic functions of anti-tumor immune cells (Pardoll, 2012), consequently promoting accelerated tumor progression. This may be one of the main reasons for the poor prognosis in these patients. If immune checkpoint blockade (ICB) therapy is administered to these patients with high expression of ICIs, it is expected to alleviate the inhibition of immune cells to a greater extent, potentially aquiring a better anti-tumor immune response. The abundant infiltration of immune cells but higher expression of ICBs might be the cause of poor prognosis of patients with higher PR scores. Administrating ICBs to alleviate immunosuppression might bring these patients unexpected benefits.

Pyroptosis has a strong association with chemotherapy, although the mechanism remains unclear. Pyroptosis induced by paclitaxel and cisplatin, the classic chemotherapeutic drugs, inhibits tumor cell proliferation and metastasis (Zhang et al., 2019). The transition from apoptosis to pyroptosis could be induced by chemotherapeutic drugs, such as mitoxantrone, cisplatin, and etoposide (Guo et al., 2021; Yu et al., 2019; Wang et al., 2017). The analysis of the target genes of classic drugs of CRC showed differential expression as the PR score increased, indicating the disparity of responses to classic therapies. The differences of response rate of nCRT in our sequencing set revealed the value of the signature on cilinical application.

## Conclusions

In conclusion, this study provides a better understanding of pyroptosis in CRC, and we developed a reliable prognostic signature based on the PRGs. Subsequent bioinformatic analyses demonstrated the association of this signature with immunotherapy, chemotherapy, and targeted molecular therapy. The signature is of value for prognostic prediction and effective personalized treatment of patients with CRC. Whereas, a multicenter clinical trial with a larger sample size is required for further validation.

## Supplemental Information

10.7717/peerj.16631/supp-1 Supplemental Information 1 Supplementary Figures.

10.7717/peerj.16631/supp-2 Supplemental Information 2 Basic information of the samples from the public databases.

10.7717/peerj.16631/supp-3 Supplemental Information 3 Pyroptosis related genes.

10.7717/peerj.16631/supp-4 Supplemental Information 4 Our RNA-sequencing data from Fudan University Shanghai Cancer Center.

10.7717/peerj.16631/supp-5 Supplemental Information 5 R code.

## Additional Information and Declarations

### Data Availability

The following information was supplied regarding data availability: The raw data are available in the Supplemental Files.
