# Supplementary material for: A pyroptosis-related signature in colorectal cancer: exploring its prognostic value and immunological characteristics
Source: PeerJ. 2023 Dec 19;11:e16631. doi: 10.7717/peerj.16631 (PMC10740607; doi:10.7717/peerj.16631)

Figure S1.
A. The profile of classic characteristics of the two clusters based on hallmarker genesets
B. Gene enrichment analysis for PRGs based on immune geneset from the GO database

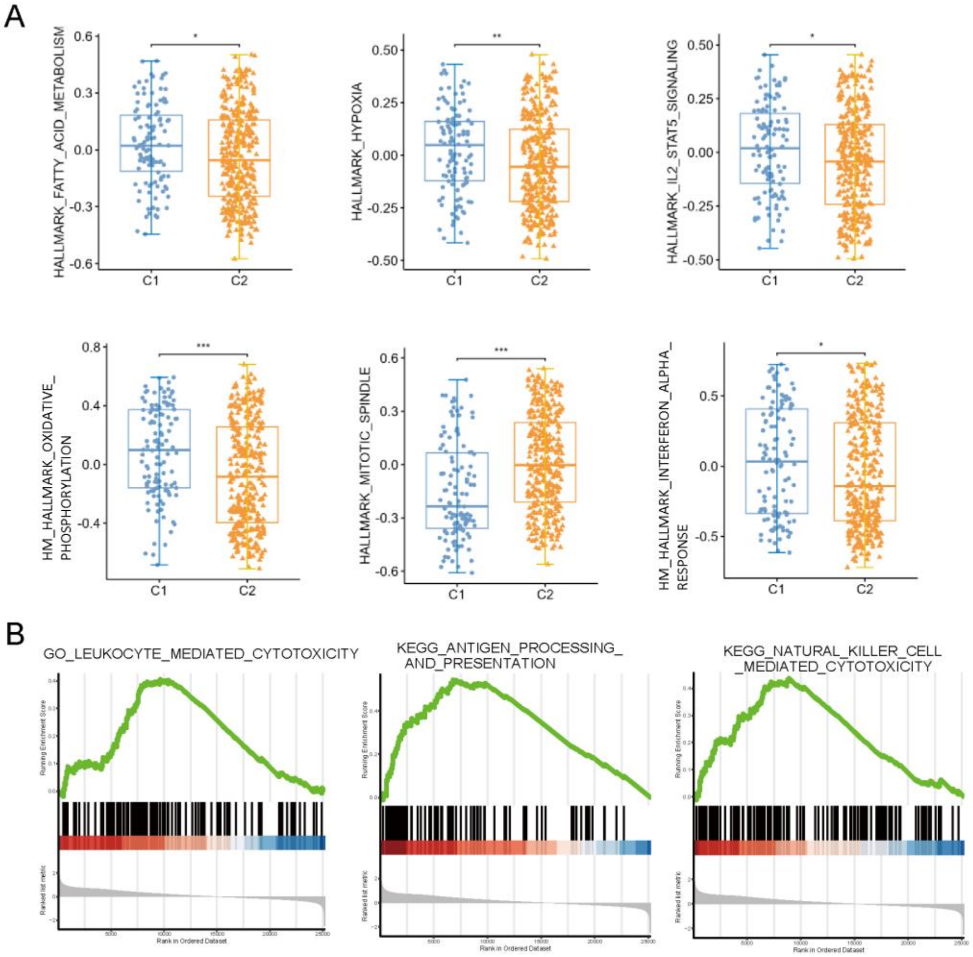

Figure S2.Multivariate cox regression analysis of OS for each key gene.

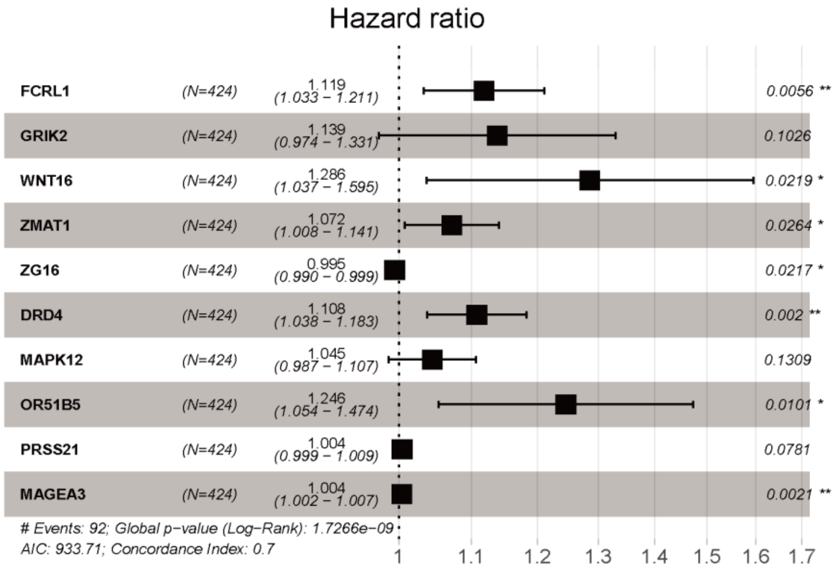

Figure S3.The correlation of immune cells and PR score based on TCIA

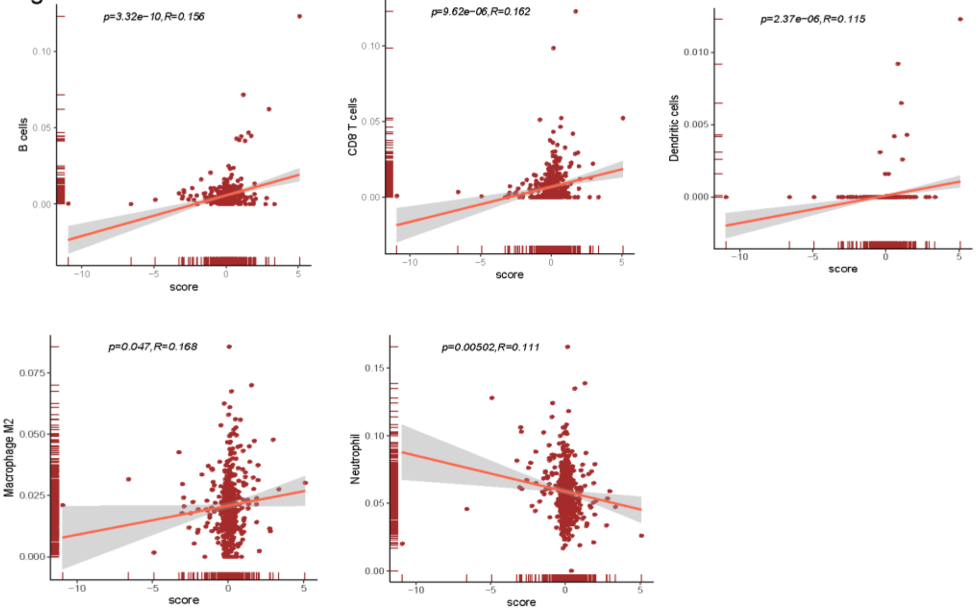

Figure S4 The differences of steps in immune cycle in two groups.

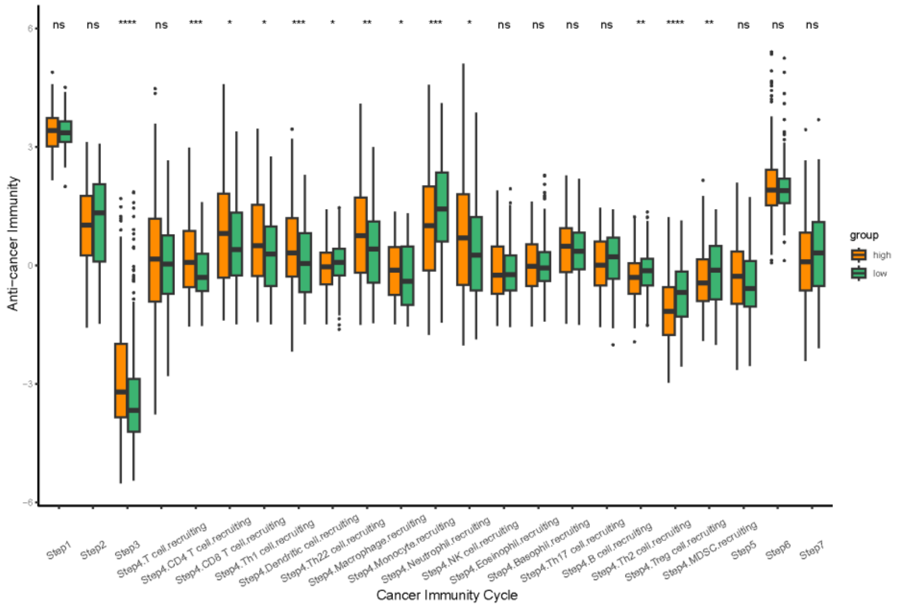

Supplement: Supplemental Information 1 [file peerj-11-16631-s001.pdf]
