# Supplementary material for: A pyroptosis-related signature in colorectal cancer: exploring its prognostic value and immunological characteristics
Source: PeerJ. 2023 Dec 19;11:e16631. doi: 10.7717/peerj.16631 (PMC10740607; doi:10.7717/peerj.16631)
Supplement: Supplemental Information 2 [file peerj-11-16631-s002.docx]

Table S1. Basic information of the samples from the public databases.

|  | TCGA-COADREAD  (n=424) | GSE39580  (n=177) |
| --- | --- | --- |
| gender |  |  |
| male | 228 (53.8%) | 96 (54.2%) |
| female | 196 (46.2%) | 81 (45.8%) |
| Age (median, quantile) | 69 (58-77) | 66 (57-75) |
| T stage |  |  |
| T1 | 10 (2.3%) | - |
| T2 | 74 (17.5%) | - |
| T3 | 290 (68.4%) | - |
| T4 | 50 (11.8%) | - |
| N stage |  |  |
| N0 | 253 (59.7%) | - |
| N1 | 97 (22.9%) | - |
| N2 | 74 (17.4%) | - |
| M stage |  |  |
| M0 | 315 (74.3%) | - |
| M1 | 58 (13.7%) | - |
| Mx | 51 (12.0%) | - |
| stage |  |  |
| I | 73 (17.2%) | 24 (13.6%) |
| II | 165 (38.9%) | 57 (32.2%) |
| III | 117 (27.6%) | 57 (32.2%) |
| IV | 58 (13.7%) | 39 (22.0%) |
| NA | 11 (2.6%) | 0 |
